# Biomolecular Interfaces in Targeted Nano-Drug Delivery: Molecular Recognition, Signaling Modulation, and Translational Pathways

**DOI:** 10.3390/biom16050722

**Published:** 2026-05-14

**Authors:** Zeyu Wang, Lixia Dai, Zhen Zhu, Xiaofei Shang

**Affiliations:** 1 Key Laboratory of New Animal Drug Project, Gansu Province, Key Laboratory of Veterinary Pharmaceutical Development of Ministry of Agriculture and Rural Affairs, Lanzhou Institute of Husbandry and Pharmaceutical Sciences, Chinese Academy of Agricultural Sciences, Lanzhou 730050, China; wzy20010421@163.com (Z.W.); 1073323010065@st.gsau.edu.cn (L.D.); 2 School of Life Sciences and Food Engineering, Hebei University of Engineering, Handan 056038, China

**Keywords:** nanocarrier materials, targeted nano-drug delivery, stimuli-responsive nanomaterials, biomimetic nanocarriers

## Abstract

Traditional pharmacotherapy is often constrained by suboptimal bioavailability and systemic toxicity. Biomolecularly inspired nano-drug delivery systems (nano-DDS) have emerged as precise platforms to overcome these barriers by orchestrating molecular interactions at the bio-nano interface. This review systematically evaluates the molecular recognition mechanisms and biochemical principles governing nano-DDS performance. We systematically evaluate how passive targeting relies on the EPR effect—dictated by the nanocarrier’s physicochemical properties—and how active targeting exploits ligand-receptor affinity to enhance cellular uptake. Special emphasis is placed on bioresponsive strategies that utilize pathological cues—such as pH gradients, redox potential, and enzymatic activity—for intelligent, on-demand drug release. Furthermore, we discuss structure-function relationships in lipid, polymeric, and biologically derived systems, highlighting their roles in modulating therapeutic signaling in oncology and inflammatory diseases. Finally, translational hurdles and emerging AI-driven molecular design strategies are critically examined.

## 1. Introduction

Traditional drug administration methods, such as oral and injectable routes, often suffer from intrinsic limitations including low bioavailability, large fluctuations in blood drug concentration, and slow onset of action [1]. These drawbacks frequently result in non-specific distribution, dose-dependent toxicity, and the need for frequent dosing, ultimately constraining therapeutic efficacy [2,3]. Furthermore, the intrinsic limitations and non-specific distribution of conventional formulations underscore an urgent need for advanced, mechanism-driven delivery systems [4,5].

Recent advances in nanomedicine have enabled the development of innovative nano–drug delivery systems (nano-DDS) based on diverse platforms, including natural product-derived nanoparticles, dendrimers, liposomes, metal-based nanoparticles, and biomimetic constructs [6,7,8]. These nanocarriers offer distinct advantages such as enhanced stability, improved solubility, and controlled drug release, collectively leading to superior tissue-specific targeting and therapeutic outcomes [7,9]. By integrating targeted recognition with stimuli-responsive release, nano-DDS provides a versatile framework for precision therapy, bridging the gap between conventional drug administration and intelligent, material-driven delivery strategies.

Nanocarriers exploit unique physicochemical properties at the 1–100 nm scale, enabling protection of therapeutic agents from premature degradation, modulation of pharmacokinetics, and spatiotemporal control over drug release [10,11,12,13]. Examples include virus-mimicking nanoparticles (CVSN), which reduce hepatic and renal accumulation, and mesoporous silica nanoparticles (MSN), which stabilize encapsulated drugs and improve delivery of hydrophobic molecules [14,15]. Prodrug-based nano-complexes have further demonstrated extended circulation times, with half-lives increased by over 12-fold relative to free drugs [15].

**Figure 1:**
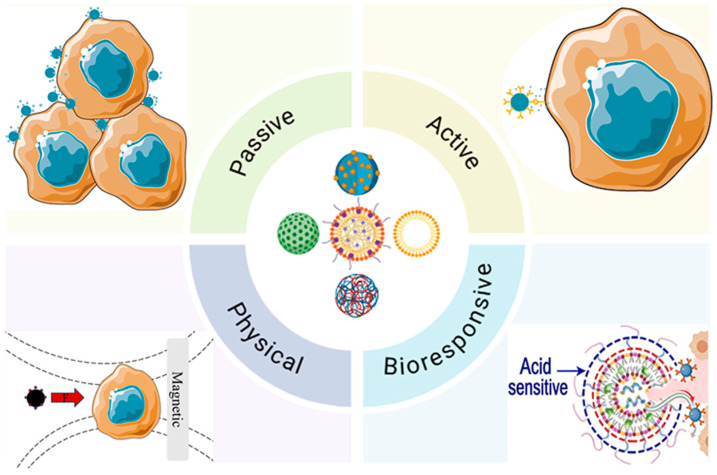
Nano-delivery systems in targeted drug delivery (Created with Adobe Illustrator 2023).

Targeted nano-DDS leverage material design and surface functionalization to achieve precise tissue and cellular recognition. Reduction-sensitive prodrug nanoparticles (SNPMTO/Cur) and laser-responsive liposomal formulations (NPs-Lip@PTX/CyA/Ce6) exemplify spatiotemporally controlled release capable of overcoming multidrug resistance [16,17]. Ligand-modified carriers, such as folic acid-functionalized ZIF-8 or dual pH/glutathione-sensitive polymeric nanogels, enhance active targeting and enable synergistic therapeutic modalities [18,19,20]. Multifunctional integration further advances nano-DDS toward theranostic applications, as demonstrated by dual thermal/acid-responsive CIPP nanoparticles for combined chemo-photothermal therapy [21,22]. Biocompatible and environmentally responsive materials, including chitosan-pectin nanocarriers, allow intelligent, flora-triggered drug release, illustrating the potential for precision and personalized interventions [23,24,25]. These advances highlight how materials-driven strategies underpin targeted nano-drug delivery, providing a robust foundation for rational design, mechanistic understanding (Figure 1), and translational development of next-generation nano-DDS.

While numerous reviews have extensively cataloged the development of targeted nano-drug delivery systems, many primarily focus either exclusively on materials synthesis or purely on broad clinical outcomes. The unique contribution and motivation of this review lie in systematically bridging the gap between materials engineering and dynamic biomolecular interactions at the bio-nano interface. Furthermore, this review provides a forward-looking perspective on how emerging AI-driven designs and pathogen-inspired bio-responsive strategies are addressing the most pressing translational and regulatory hurdles. By systematically connecting molecular recognition principles with structural functionalization, this review offers a comprehensive roadmap for the rational design of the next generation of precision nanotherapeutics.

## 2. Targeted Nano-DDS: Mechanisms and Materials Design Principles

### 2.1. Passive Targeting: Delivery Optimization Based on the EPR Effect

**Figure 2:**
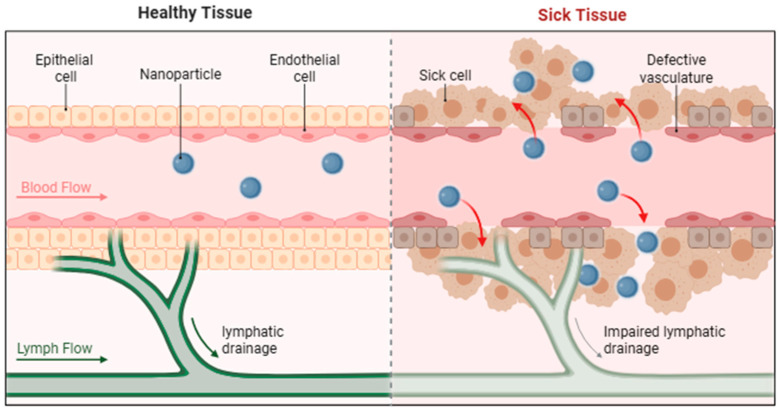
Passive targeting: Delivery optimization based on the EPR Effect (Adapted from Biorender.com, created with Adobe Illustrator).

Nanocarrier size critically influences tumor accumulation efficiency. Mahmoud et al. [26] systematically correlated lipid nanoparticle (LNP) dimensions with targeting efficacy in hepatocellular carcinoma (HCC), identifying ~150 nm as the optimal size for tumor enrichment (Figure 2). Characterization via dynamic light scattering (DLS) and transmission electron microscopy (TEM) demonstrated that this size range allows traversal of tumor endothelial gaps (mean pore size ~400 nm) while avoiding rapid renal clearance (threshold < 5.5 nm) [27]. Compared with free doxorubicin, size-optimized LNPs increased tumor drug concentration threefold (*p* < 0.01) and reduced cardiotoxicity by 62% (assessed via creatine kinase-MB [CK-MB] levels) [26]. The enhanced permeability and retention (EPR) effect, however, exhibits substantial tumor-type variability. To overcome this heterogeneity, Kinoshita et al. (2021) [28] developed a human serum albumin dimer (HSA-d) system exploiting albumin’s intrinsic “stealth” properties (hydration radius ~7 nm) and gp60 receptor-mediated transcytosis. In pancreatic ductal adenocarcinoma (PDAC) with low vascular density, this approach increased drug accumulation by 50% [28]. Isotope tracing confirmed a 2.3-fold prolongation of retention time in low-permeability tumors (K_trans_ < 0.1 min^−1^) relative to conventional nanoparticles (*p* < 0.05), highlighting a viable strategy to address tumor heterogeneity [28].

Beyond size optimization, precise modulation of nanocarrier surface properties is essential for effective passive targeting. Polyethylene glycol (PEG) modification, the current gold standard, extends circulation half-life 5- to 10-fold by forming a hydration layer that minimizes recognition by the reticuloendothelial system (RES) [29]. Nevertheless, excessive PEGylation can hinder cellular uptake, a phenomenon known as the “PEG dilemma.” To mitigate this, Novio et al. engineered nanostructured coordination polymers (NCPs) featuring gradient PEGylation (15% surface density) and hierarchically porous architectures (pore size 20–50 nm) via tunable Zn^2+^ coordination [30]. This design maintained stealth properties during circulation (plasma protein adsorption < 5%) while enabling tumor-triggered pore expansion for drug release. In orthotopic breast cancer models, these NCPs achieved a tumor penetration depth of 300 μm (vs. 80–100 μm for conventional nanoparticles) and a 2.8-fold increase in the drug distribution uniformity index (DUI) [30].

Despite these advances, elevated tumor interstitial fluid pressure (IFP; 40–100 mmHg) and dense extracellular matrix (ECM) remain significant barriers to effective delivery [31]. Recent evidence indicates that nanocarrier shape anisotropy can enhance tumor penetration [32]. When combined with matrix-modulating strategies, such as hyaluronidase pretreatment or TGF-β inhibition, penetration depth can be further improved by 40–50% [31,32].

### 2.2. Active Targeting: Ligand-Receptor Recognition and Intracellular Signaling

**Figure 3:**
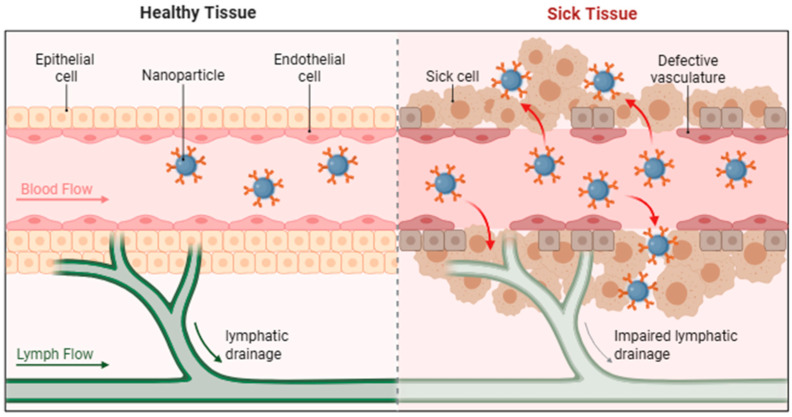
Active targeting: Precise recognition mediated by ligands (Adapted from Biorender.com, created with Adobe Illustrator).

Active targeting strategies involve functionalizing nanocarrier surfaces with specific ligands to enable recognition and binding to receptors overexpressed on target cells (Figure 3), thereby achieving precise drug delivery [33,34]. Compared with passive targeting, active targeting can markedly enhance accumulation at the disease site while minimizing off-target toxicity [34,35].

Ligand–receptor interactions form the mechanistic basis of active targeting. Monoclonal antibodies can selectively bind HER2 receptors overexpressed in tumor cells [36], short peptides target integrin αvβ3 in tumor neovasculature [37], and polysaccharides recognize CD44 receptors on diverse malignancies [38]. These ligands are conjugated to nanocarrier surfaces, such as liposomes or polymeric nanoparticles, via covalent chemistry or physical adsorption, endowing nanocarriers with active targeting functionality [39,40].

Recent studies highlight the therapeutic potential of ligand-modified systems. Li et al. [41] developed ANGPT2 peptide-functionalized PEGylated liposomes targeting the glioma vascular marker ANGPT2. This formulation exhibited a four-fold increase in tumor uptake relative to unmodified liposomes (*p* < 0.01) and reduced orthotopic glioma volume by 70% via VEGF pathway inhibition, demonstrating both effective targeting and improved penetration across the blood–brain barrier. Similarly, Al-Shadidi et al. [42] applied folic acid (FA)-modified chitosan nanoparticles (FA-CS-NPs) for breast cancer therapy. FA-CS-NPs achieved ~90% cellular uptake in folate receptor (FRα)-expressing cancer cells, compared with <20% for unmodified nanoparticles, while preserving >85% viability of normal breast epithelial cells (MCF-10A), highlighting an enhanced therapeutic index.

Despite these advantages, clinical translation of active targeting remains challenging, with ligand stability being a critical limitation [43]. Björgvinsdóttir et al. [44] reported that antibody-conjugated liposomes experienced over 50% reduction in surface ligand density after 4 h of in vivo circulation, primarily due to competitive adsorption by serum proteins and lipid exchange. Strategies to enhance ligand stability include: (1) covalent coupling, providing superior retention compared with physical adsorption [29,45], and (2) biomimetic membrane modification, which leverages cell-membrane–mimicking structures to reduce nonspecific protein adsorption and extend ligand half-life [27,41,46]. Collectively, these studies underscore the importance of integrating ligand selection, surface chemistry, and material design to achieve robust, targeted delivery while addressing translational hurdles associated with in vivo stability.

The rational design of these active targeting systems requires a precise structural balance to maximize receptor-mediated endocytosis while preventing steric hindrance. For instance, in vitro optimization assays are critical for determining the ideal ligand density on the nanocarrier surface. When optimizing mannosylated solid lipid nanoparticles, adjusting the surface mannose density to 15–20 molecules/μm^2^ achieved high-affinity binding (Kd ~10^−8^ M), which enhanced intracellular delivery by over threefold compared to unmodified particles. Subsequent in vivo models confirmed that such rationally designed systems not only significantly increase target tissue accumulation but also drastically reduce systemic toxicity. For example, specific ligand modifications have been shown to preserve normal epithelial cell viability (>85%) while achieving ~90% cellular uptake in malignant cells, proving the essential role of precise biomolecular engineering in overcoming biological barriers.

### 2.3. Physical Targeting: Spatiotemporal Controlled Release Guided by External Stimuli

**Figure 4:**
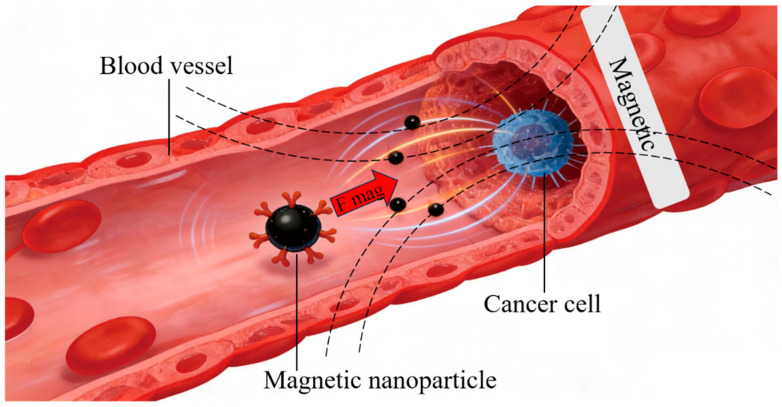
Physical targeting: Magnetic-responsive delivery system (Adapted from [36], created with Adobe Illustrator).

Physical targeting strategies utilize external stimuli, such as light, magnetic fields (Figure 4), temperature, or ultrasound, to achieve controllable drug release and precise site-specific delivery [47]. Compared with passive or ligand-mediated targeting, physical targeting offers distinct advantages, including spatiotemporal precision, tunable responsiveness, and remote control over drug release [48]. In recent years, these approaches have demonstrated considerable potential in oncology, anti-inflammatory therapy, and neurodegenerative disease management [49,50,51].

Light-responsive delivery systems release therapeutic agents upon exposure to specific wavelengths (UV, visible, or near-infrared), providing high spatial and temporal resolution with minimal invasiveness [52,53]. Lee et al. developed photosensitive nanoparticles (PS-NPs) incorporating surface-bound photosensitizers and chemotherapeutic drugs encapsulated in the core. Upon near-infrared irradiation, PS-NPs generate reactive oxygen species (ROS) at tumor sites, inducing structural disintegration and controlled drug release. Compared with conventional pH-responsive systems, this approach achieved an 80% higher release rate while reducing systemic toxicity, including a 60% decrease in cardiotoxicity in murine models. Moreover, photothermal effects synergistically enhanced tumor cell membrane permeability, facilitating intracellular drug uptake [53].

Magnetic-responsive delivery systems typically employ superparamagnetic iron oxide nanoparticles (SPIONs) or magnetic liposomes to enrich drug concentrations at lesions under external magnetic guidance [54,55]. For example, Jingyi et al. designed drug-loaded magnetic liposomes (MLs) encapsulating Fe_3_O_4_ nanoparticles and oxaliplatin, targeting colorectal cancer lymph node metastases. External magnetic guidance enhanced ML accumulation in metastatic lymph nodes fourfold relative to passive targeting, prolonged retention beyond 24 h, increased treatment efficiency to 80%, and reduced off-target liver and kidney toxicity (ALT/AST decreased by 45%) [55]. Additionally, alternating magnetic fields induced localized hyperthermia, triggering tumor cell apoptosis and enabling synergistic chemo-hyperthermia therapy [56,57].

Beyond light and magnetic stimuli, temperature- and ultrasound-responsive systems have shown promising capabilities [58,59]. Noel et al. developed a focused ultrasound (FUS)-triggered nanoemulsion that reversibly opened the blood–brain barrier via mechanical stress, enhancing delivery of the anti-Aβ antibody for Alzheimer’s disease by 3.5-fold [60]. Collectively, these studies highlight how stimuli-responsive nanomaterials can be rationally engineered to achieve precise, spatiotemporally controlled drug release, illustrating the potential of physical targeting to overcome biological barriers while minimizing systemic side effects.

### 2.4. Bioresponsive Targeting: Intelligent Release Driven by the Microenvironment

**Figure 5:**
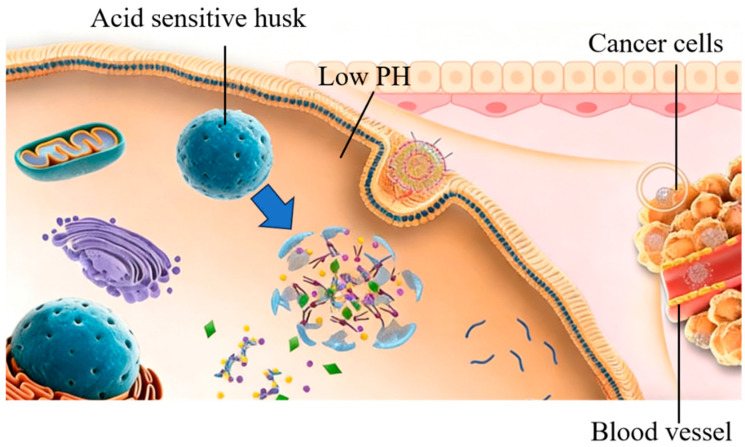
Bioresponsive targeting: PH-responsive delivery system (Adapted from [65], created with Adobe Illustrator).

Bioresponsive targeting leverages unique biochemical and physiological cues within the tumor microenvironment (TME), including acidic pH, elevated enzyme activity, and hypoxia (Figure 5), to trigger site-specific drug release [61,62]. Tian et al. developed pH-sensitive liposomes that rapidly released elemene (ELE) within the acidic TME, resulting in a twofold enhancement of antitumor efficacy compared with conventional formulations and reversal of multidrug resistance (MDR) [63]. Similarly, Sun et al. engineered glutathione (GSH)-responsive nanocarriers in which disulfide bond cleavage under high TME GSH levels enabled controlled triptolide (TP) release, broadening the therapeutic window by a factor of three relative to traditional therapy [64].

Current research increasingly emphasizes the integration of multiple targeting strategies to overcome the limitations of single-mechanism approaches [37,66]. Rana et al. reported a “smart” delivery system combining active targeting via HER2 antibody modification with an MMP-2 enzyme-triggered stimuli-responsive release, achieving approximately 95% tumor inhibition in breast cancer models [37]. Qiao et al. combined paclitaxel-loaded nanocarriers with immune checkpoint inhibitors to enhance T-cell infiltration in the TME, extending survival by 40% in triple-negative breast cancer models [66].

Despite these promising advances, clinical translation of bioresponsive nano-DDS remains challenging. Key obstacles include long-term biocompatibility and safety of nanomaterials, scalability and reproducibility of production processes, and patient-specific variability arising from tumor heterogeneity [37]. Future directions emphasize AI-assisted nanocarrier design, development of green and sustainable materials, and personalized delivery strategies tailored to individual patient pathology [26,42]. Collectively, bioresponsive targeting illustrates the potential of intelligent, microenvironment-sensitive nanomaterials to achieve precise, on-demand drug release, highlighting the synergy between material design, therapeutic efficacy, and translational applicability.

## 3. Nanocarrier Materials: Structure–Function–Biointeraction Relationships

While Section 2 outlined the underlying biological mechanisms and external stimuli driving active, passive, and bioresponsive targeting, the successful realization of these mechanisms is fundamentally dictated by the physical and chemical nature of the nanocarrier materials themselves. This section specifically examines the structure-function-biointeraction relationships of distinct material classes—spanning lipid-based, polymeric, inorganic, and biologically derived systems. We detail how intrinsic architectural features, such as matrix crystallinity, polymer degradation kinetics, and inorganic porosity, directly govern drug encapsulation efficiency, in vivo stability, and the ultimate biological fate of the therapeutic payload.

### 3.1. Lipid-Based Nanosystems

Polyethylene glycol (PEG) modification is a well-established strategy to extend liposome circulation and evade clearance by the mononuclear phagocyte system (MPS) [41,67]. PEG chains form a hydrophilic steric barrier on the liposome surface, reducing plasma protein adsorption and subsequent macrophage recognition. For example, L. Li et al. developed a PEGylated doxorubicin liposome (PLD variant) by optimizing PEG chain length (2 kDa) and surface density (5 mol%), which increased circulation half-life from <2 h to ~12 h in glioma models [41]. Coupled with precise control of particle size (<60 nm) and lipid composition (DSPC/cholesterol/PEG-DSPE), this design enhanced blood–brain barrier (BBB) penetration, increasing doxorubicin accumulation in brain tumors by 40% [68].

Similarly, Yuan et al. reported an enzyme-responsive PEGylated liposome (PEG_2.5_K-MLP-L) incorporating an MMP-2-sensitive peptide linker in the PEG layer, enabling tumor microenvironment-triggered drug release [68]. In colorectal cancer models, this system improved drug deposition in primary tumors and metastatic lymph nodes via lymphatic drainage, increasing the 90-day survival rate from 45% to 80%, demonstrating the synergistic advantage of combining PEGylation with stimuli-responsive design for enhanced tumor-targeted therapy. Despite these benefits, PEGylation faces translational challenges. Bjorgvinsdottir et al. observed that antibody ligands (e.g., anti-EGFR scFvs) conjugated via PEG-DSPE lost >50% surface density within 4 h in vivo, reducing targeting efficiency. This loss was primarily attributed to lipid exchange, specifically the transfer of the DSPE anchor to high-density lipoproteins (HDL) in circulation [44].

#### 3.1.1. Ligand-Modified Liposomes

The hallmark of targeted drug delivery lies in achieving precise accumulation in diseased tissues via ligand–receptor recognition [69]. Ligand-modified nanocarriers enhance delivery efficiency through multiple mechanisms: (i) increasing binding affinity to target cells; (ii) promoting membrane fusion or endocytosis; and (iii) modulating signaling pathways to exert synergistic therapeutic effects [70,71]. This multi-mechanistic synergy positions ligand modification as a key strategy to overcome biological barriers and achieve precise therapy [72,73].

In anti-parasitic applications, Khosravi et al. developed mannosylated solid lipid nanoparticles (PM-SLN-M) targeting the mannose-binding protein-1 (MBP-1) receptor on *Toxoplasma gondii* [74]. Optimizing surface mannose density to 15–20 molecules/μm^2^ achieved high-affinity binding (K_d_ ~10^−8^ M), enhancing intracellular praziquantel delivery to 82.3% versus 27.4% for unmodified particles, and tripling anti-parasitic efficacy. Importantly, systemic toxicity was markedly reduced: HEK293 cell survival exceeded 85% for PM-SLN-M, compared to 23% for free drug (*p* < 0.001), demonstrating that ligand density optimization is critical for efficient and low-toxicity delivery [74]. For central nervous system applications, Li et al. designed TREM2-targeting peptide-modified PEGylated liposomes (peptide-PLD) based on glioma microenvironment characteristics [75]. A high-affinity targeting peptide identified via phage display was site-specifically conjugated to the liposome surface (grafting rate 92%). In vivo two-photon imaging revealed 6.8-fold higher BBB penetration than unmodified liposomes, with tumor accumulation rising from 1.6% to 11.2% ID/g. Mechanistic studies showed the system downregulated the Akt/GSK3β/β-catenin axis via TREM2-mediated endocytosis: p-Akt (Ser473) decreased by 72%, and nuclear β-catenin translocation decreased by 68%. These dual effects reduced U87MG cell migration by 70% and extended median survival in glioma-bearing mice from 28 to 49 days (*p* < 0.001), highlighting the integration of delivery and signaling modulation in rationally designed ligand-modified systems [75].

Despite these advantages, challenges remain: (i) precise control of ligand density and orientation, as conventional conjugation may impair bioactivity; (ii) masking of ligands by the in vivo protein corona, reducing targeting efficiency; and (iii) limited design principles for multi-target cooperative recognition. Emerging strategies, including computer-aided ligand design (e.g., AlphaFold for binding interface prediction), site-specific conjugation via non-natural amino acids, and stimuli-responsive stealth-to-targeting conversion, offer potential solutions to these challenges [76,77].

#### 3.1.2. Solid Lipid Nanoparticles

Solid lipid nanoparticles (SLNs) represent a new generation of nanocarriers (Table 1), offering distinct advantages owing to their ordered solid lipid matrix [78]. This architecture addresses key limitations of conventional dosage forms, particularly for poorly soluble drugs, by enabling high drug loading and controlled release [79]. The solid lipid matrix provides a more organized molecular environment than liquid carriers, improving drug encapsulation efficiency and stabilizing the payload [80,81]. Khosravi et al. demonstrated that albendazole-loaded SLNs achieved 3–5-fold higher drug loading than conventional formulations, maintaining physical stability for up to six months in simulated gastrointestinal fluid. DSC and XRD analyses confirmed the amorphous dispersion of drug molecules within the lipid matrix, enhancing dissolution and bioavailability [74]. Agrawal et al. introduced the “lipid–drug affinity matching” principle, showing that tuning lipid chain length, charge, and hydrophobicity (logP) can increase loading efficiency above 90%. High-melting-point lipids, such as glyceryl tristearate (≥55 °C), minimized drug leakage (<5%), ensuring long-term storage stability [82].

SLNs also provide controlled release benefits. In simulated intestinal fluid, albendazole SLNs exhibited a biphasic release: ~30% released within the first 0–8 h, followed by sustained zero-order release over 60 h. Compared to free drug, the release rate was reduced by 50%, extending the antiparasitic action window from 12 to 72 h and reducing dosing frequency from thrice to once daily [74]. Nevertheless, challenges remain for clinical translation: (i) achieving batch-to-batch consistency at scale; (ii) maintaining stability during freeze-drying and re-dispersion; and (iii) correlating in vivo degradation kinetics with pharmacodynamics [86,87,88]. Microfluidic production technologies offer promising solutions, allowing particle size control within 5% coefficient of variation and increasing production efficiency 20-fold [89].

#### 3.1.3. Sustained Release and Stability Advantages of Nanostructured Lipid Carriers

Nanostructured lipid carriers (NLCs) improve upon traditional SLNs by incorporating blends of solid and liquid lipids, offering enhanced drug loading flexibility and controlled release performance [90,91]. Sui et al. developed lipid prodrug liposomes combining enzyme-responsive release with immunomodulatory functions, which reprogrammed neutrophil phenotypes to enhance T-cell infiltration in the tumor microenvironment, resulting in a 60% reduction in tumor volume in colorectal cancer models [77]. Similarly, Li et al. conjugated ANGPT2-specific peptides to PEGylated liposomes, achieving integrated targeting for glioma imaging and therapy, markedly improving diagnostic and therapeutic efficiency relative to conventional liposomes [75].

Overall, lipid-based nanocarriers, particularly liposomes and NLCs, have advanced nanomedicine efficacy and safety through multiple strategies: long-circulation modification, ligand-mediated targeting, optimization of lipid composition for enhanced stability, and stimuli-responsive drug release [75,82]. Despite these advances, clinical translation remains constrained by ligand stability, large-scale manufacturing challenges, and the need for patient-specific customization [92]. Future directions emphasize novel ligand anchoring strategies, AI-driven lipid design, and development of multi-modal theranostic platforms to enable personalized and precise interventions [68,77,93].

### 3.2. Polymer Nanoparticles

#### 3.2.1. Synthetic Biodegradable Polymers: PLGA-Based Delivery Systems

Biodegradable polymers, owing to their tunable physicochemical properties and excellent biocompatibility, have emerged as versatile platforms for modern drug delivery [94]. Among them, poly lactic-co-glycolic acid (PLGA) and chitosan are widely utilized for targeted delivery applications [95,96,97] (In Table 2). PLGA, an FDA-approved degradable polymer, allows precise control over degradation kinetics from weeks to months by adjusting the lactic-to-glycolic acid ratio [98,99]. Its hydrolytic degradation products, lactic acid and glycolic acid, enter the tricarboxylic acid cycle, ensuring minimal systemic toxicity [100,101,102].

Targeted modifications further improve polymer-based delivery efficiency. For instance, Mukerjee et al. engineered PLGA nanoparticles conjugated with a monoclonal antibody against the *Cryptosporidium*-specific CP2 protein. Compared to unmodified PLGA nanoparticles, antibody-conjugated NP-906 achieved a 12.7-fold higher uptake in *Cryptosporidium*-infected Caco-2 cells, a 78-fold enhancement in in vivo anti-parasitic efficacy, and reduced off-target accumulation in the liver and spleen [103]. This study highlights the importance of precise surface functionalization for pathogen-targeted therapy. Chitosan’s intrinsic pH responsiveness and cationic nature have been exploited for gastrointestinal disease management. In DSS-induced colitis models, IOCS/5-ASA reduced disease activity index by 58.7%, alleviated colon shortening by 62.3%, and improved histological scores 4.3-fold compared to free 5-ASA. MRI tracking of iron oxide nanoparticles enabled integrated treatment-monitoring, correlating T2-weighted signal intensity with inflammation severity [25].

#### 3.2.2. Natural Polysaccharides: Chitosan and Guar Gum-Based Systems

Beyond synthetic options, naturally derived polymers like chitosan and guar gum are extensively exploited for localized and mucosal delivery. Chitosan, a naturally derived cationic polysaccharide, exhibits strong mucoadhesive properties and electrostatic interactions with negatively charged cell membranes, substantially enhancing cellular uptake of encapsulated therapeutics [98,104,105]. Its intrinsic pH responsiveness and cationic nature have been particularly advantageous for gastrointestinal disease management. Zhang et al. developed a chitosan-based composite nano-system (IOCS/5-ASA) for colitis, achieving: (1) enhanced 5-aminosalicylic acid (5-ASA) loading (82.3 ± 3.5%) via complexation with iron oxide nanoparticles; (2) pH-dependent colonic release mediated by an Eudragit S100 coating; and (3) improved intestinal barrier integrity through chitosan-mediated mucoadhesion and barrier repair. Guar gum, another natural polymer, serves as a colon-targeting carrier through enzymatic degradation by colonic microbiota [106,107]. Strategies such as cross-linking, graft modification, and nanoscale processing (150–300 nm) optimize controlled release and mucosal interaction. Building on these principles, Niu et al. designed a triple-targeted chitosan-coated liposome (Spore-LP/CS) for colorectal cancer therapy, integrating: (1) *Clostridium difficile* spore conjugation for microbial targeting; (2) pH-responsive dissolution of chitosan/Eudragit at colonic pH; and (3) enzyme-triggered release by colonic enzymes. In CT26 tumor models, this system enhanced oxaliplatin/anti-PD-L1 efficacy, increasing tumor inhibition 2.1–3.3-fold, CD8^+^ T cell infiltration 4.8-fold, and reducing systemic toxicity relative to monotherapies [108]. Collectively, biodegradable polymers, including synthetic and natural variants, offer versatile, biocompatible platforms for targeted and stimuli- responsive drug delivery, establishing a foundation for next-generation nanotherapeutics [109].

**Table 2:** Performance and targeting mechanisms of polymer-based nanocarriers.

Nanoparticle Type	Targeting Strategy	Biological Model	Key Outcomes	Ref.
PLGA Nanoparticles	Active (Monoclonal antibody against CP2)	Cryptosporidium-infected Caco-2 cells (in vitro and in vivo)	12.7-fold higher cellular uptake; 78-fold enhancement in in vivo anti-parasitic efficacy.	[103]
Iron Oxide-Chitosan Nanocomposites	pH-responsive (Eudragit S100) and Mucoadhesive	DSS-induced ulcerative colitis (in vivo)	Disease activity index reduced by 58.7%; histological scores improved 4.3-fold.	[25]
Chitosan-coated Liposomes	Triple-targeted: Microbial spores, pH, and Enzyme	CT26 colorectal tumor (in vivo)	Tumor inhibition increased 2.1–3.3-fold; CD8+ T cell infiltration increased 4.8-fold.	[108]

### 3.3. Inorganic Nanoparticles

#### 3.3.1. Stimuli-Responsive Metal Nanocarriers

Metal-based nanomaterials have recently emerged as promising platforms for intelligent drug delivery due to their tunable physicochemical properties, including controllable size and morphology, abundant surface chemistry, and sensitivity to external stimuli such as pH, temperature, enzymes, and light [110]. These attributes enable the rational design of nanocarriers capable of controlled and targeted drug release, thereby enhancing therapeutic efficacy while minimizing systemic toxicity [111] (In Table 3). Chen et al. developed a poly(ethylene glycol) methacrylate (PEGMA)-modified bimetallic Prussian blue analog drug delivery system (PBA-DDSs) that exploits the pH difference between tumor tissues (pH 6.5–7.0) and normal tissues (pH 7.4) for selective drug release [112]. Under tumor-mimicking pH conditions (pH 6.5), doxorubicin release reached 75.9%, significantly higher than that under physiological pH. In vitro studies showed a 25% enhancement in MCF-7 breast cancer cell growth inhibition compared to free doxorubicin. Following AS1411 aptamer-mediated active targeting, in vivo experiments demonstrated a 3.2-fold increase in tumor accumulation and a 58% reduction in cardiac drug distribution, effectively mitigating doxorubicin-associated cardiotoxicity. This study highlights the advantages of metal-based nanocarriers in addressing key limitations of conventional chemotherapeutics, including poor targeting and systemic toxicity.

In oral delivery applications, metal–organic frameworks (MOFs) have attracted significant attention due to their tunable pore structures and facile surface functionalization [113,114]. Raza and Wu demonstrated that by adjusting pore size (2–10 nm) and surface functional groups, MOF-based carriers can achieve dual pH- and enzyme-responsive drug release. Under simulated gastric conditions, drug release was <10% within 2 h, protecting acid-sensitive therapeutics. In contrast, intestinal pH and specific enzymatic conditions triggered >80% release. Compared with conventional liposomes, these MOFs exhibited 40% higher drug loading efficiency and maintained >95% drug retention after 30 days at 37 °C. Furthermore, surface conjugation with targeting ligands enhanced drug accumulation at intestinal lesion sites by 3.5-fold in an inflammatory bowel disease model, providing a versatile strategy for oral biopharmaceutical delivery [115].

Collectively, these studies demonstrate that metal-based nanomaterials with their customizable structures, environmental responsiveness, and multifunctional capabilities effectively overcome critical challenges of traditional delivery systems, including poor tissue penetration, limited targeting, and uncontrolled release [112,113,114,115]. Future work should focus on optimizing long-term in vivo biocompatibility, scalable and reproducible synthesis, and clinical translation pathways to fully harness their potential in precision medicine [116].

**Table 3:** Stimuli-responsiveness and outcomes of inorganic nanocarriers.

Nanoparticle Type	Targeting Strategy	Biological Model	Key Outcomes	Ref.
Bimetallic Prussian Blue Analogs	Dual: pH-responsive and Active (AS1411 aptame	MCF-7 breast cancer (in vitro and in vivo)	3.2-fold increase in tumor accumulation; 58% reduction in cardiac drug distribution.	[112]
Metal–Organic Frameworks (MOFs)	Dual: pH- and Enzyme-responsive	Inflammatory bowel disease (in vivo)	40% higher drug loading efficiency; drug accumulation at intestinal lesions enhanced 3.5-fold.	[115]
Ag-Cu Alloy Nanoparticles	Non-specific/Synergistic ion release	*S. aureus* and *E. coli* biofilms (in vitro)	82.3% biofilm eradication efficiency; intracellular ROS elevated ~3.5-fold.	[117]

#### 3.3.2. Antibacterial and Anti-Inflammatory Applications of Metal Nanoparticles

Metal nanoparticles have garnered extensive attention in biomedical research due to their unique physicochemical properties and high bioactivity [118,119,120]. Among them, silver (Ag) and copper (Cu) nanoparticles are prominent for their broad-spectrum antimicrobial activity. Mechanistic studies indicate that these nanoparticles exert antibacterial effects via multiple pathways, including disruption of bacterial membrane integrity, interference with electron transport chains, induction of reactive oxygen species (ROS), and release of bioactive metal ions. Notably, alloy nanoparticles often outperform single-metal systems in antibacterial efficacy [121,122,123].

Fan et al. synthesized uniform Ag-Cu alloy nanoparticles via an advanced co-reduction method and systematically evaluated their activity against *Staphylococcus aureus* and *Escherichia coli* biofilms [117]. Nanoparticles with a 1:1 Ag:Cu molar ratio exhibited the strongest synergistic antibacterial effect. Mechanistic analysis using electron spin resonance (ESR) and inductively coupled plasma mass spectrometry (ICP-MS) revealed that this synergy arose from two primary mechanisms: (1) elevated intracellular ROS levels (~3.5-fold higher than controls), causing oxidative stress and DNA damage; and (2) synergistic release of Ag^+^ and Cu^2+^ ions, disrupting bacterial membrane potential by ~60%. Consequently, the Ag-Cu alloy nanoparticles achieved a biofilm eradication efficiency of 82.3%, markedly higher than individual Ag or Cu nanoparticles, with a minimum inhibitory concentration (MIC) as low as 1 μg/mL—substantially lower than typical clinical antibiotic doses. Beyond antimicrobial applications, metal nanoparticles have been engineered for multifunctional therapeutic delivery. Li et al. designed polyethylene glycol (PEG)- and CRGDK-targeting peptide-modified gold nanoparticles (Au NPs, 20 nm) for co-delivery of retinoic acid (RA) and miRNA-29b [124]. Using microfluidic fabrication, this nanocarrier demonstrated: (1) high drug loading efficiency (RA 8.7%, miRNA > 90%); (2) pH-responsive release, with drug release at pH 5.0 being 3.2-fold higher than at pH 7.4; and (3) an X-ray attenuation coefficient of 5.8 HU/mM, ~2.3 times that of clinically used iodine contrast agents.

Despite these advances, key challenges remain for clinical translation, including long-term biosafety concerns related to metal ion accumulation, batch-to-batch consistency during large-scale production, and stability in complex biological environments [125,126]. Future research should emphasize the development of novel surface modification strategies to enhance targeting, design of stimuli-responsive release systems for precise therapy, and establishment of standardized safety evaluation frameworks [127]. With continued integration of nanotechnology and biomedicine, metal nanoparticles are poised to achieve significant breakthroughs in both antimicrobial therapy and multifunctional therapeutic applications.

#### 3.3.3. Development of Multifunctional Metal Nanocarriers

The multifunctional design of nanocarriers, particularly metal-based systems, has emerged as a crucial strategy to address limitations in conventional drug delivery. Illustrating principles of functional integration, Zou et al. developed a porous network via self-assembly of low-viscosity alginate (LVA) and anthocyanins, which enhanced the bioavailability of cyanidin-3-glucoside (C3G) by 27.4% and exhibited a sustained-release profile compatible with intestinal absorption [128]. Focusing specifically on metal nanoparticles, Valdivieso et al. synthesized core–shell silver-gold nanoparticles (Ag-Au NPs) capable of localized high-temperature drug release via photothermal effects. In cancer models, this approach increased tumor cell apoptosis by 60% while minimizing damage to surrounding normal tissues, demonstrating the potential of multifunctional metal nanocarriers for precision therapy [129].

Despite these advances, clinical translation of multifunctional metal-based nanocarriers remains challenging. Key issues include long-term cytotoxicity of certain compositions, such as Ag-Cu alloys, and the large-scale synthesis and batch consistency of hybrid materials, including metal–organic frameworks (MOFs) [115,117]. Future directions involve integrating computational materials science with microfluidic technologies to optimize nanocarrier design, fabrication, and evaluation pipelines, as well as exploring emerging applications in gene delivery and immunomodulation [124,126].

### 3.4. Biologically Derived Nanocarriers

#### 3.4.1. Bionic Design-Driven Targeted Delivery

In Recent years, bioinspired nanosystems have garnered substantial interest in precision drug delivery due to their distinctive biomimetic properties (Table 4). By emulating morphological features or functional mechanisms of natural organisms, these systems facilitate efficient targeted delivery, overcoming limitations associated with conventional strategies. Key design principles include biomimetic morphology (e.g., cell membrane coatings, virus-like structures), biomimetic motility (e.g., bacterial chemotaxis, sperm flagellar propulsion), and biomimetic interfaces (e.g., adhesive protein modification, topological surface optimization). Collectively, these approaches enhance nanocarrier targeting, tissue penetration, and biocompatibility, thereby improving therapeutic efficacy [130,131].

As an illustrative example, Neagu et al. engineered a magnetic nanorobot that mimics sperm flagellar motion and the surface topology of spiral cyanobacteria. This system incorporated CXCR4 chemokine receptors to enable directional migration along SDF-1α gradients within the tumor microenvironment. By mechanically disrupting the ECM, the nanorobot increased doxorubicin accumulation by 50% (*p* < 0.01) in an MCF-7 tumor model compared to conventional liposomes, while markedly reducing cardiotoxicity [132].

Biomimetic strategies have also been applied to oral drug delivery, addressing challenges such as mucus layer clearance and poor epithelial permeability [136]. Sang et al. developed an integrated bioinspired system for oral insulin delivery, leveraging viral evolutionary principles for enhanced adhesion and cellular uptake [14]. Electron beam lithography generated virus-like spike structures on PLGA nanoparticles, increasing physical anchoring through expanded contact with intestinal villi. Functionally, intestinal-specific adhesive proteins were incorporated to resist shear forces in the mucus layer, resulting in an 8.3-fold increase in adhesion compared to smooth-surface particles. Additionally, pH-responsive shells were designed to trigger insulin release specifically in the ileum. This design increased insulin bioavailability from 5.2% to 15.7% (≈3-fold improvement over unmodified particles) and extended fasting blood glucose regulation to 12 h [14].

#### 3.4.2. Multifunctional Integration of Cell Membrane Biomimetic Systems

Natural cell membrane coating technology has attracted considerable attention in nanomedicine due to its superior biocompatibility and intrinsic biomimetic functions [137]. This strategy involves isolating membranes from natural cells, such as red blood cells (RBCs), white blood cells, or cancer cells, and coating them onto synthetic nanoparticles to create hybrid delivery platforms [133,138]. A representative example is red blood cell membrane-coated nanoparticles (RBC-NPs), which utilize the CD47-mediated “self-marking” mechanism to evade immune clearance [133]. CD47, a widely expressed “don’t eat me” signal protein on RBCs, interacts with signal regulatory protein α (SIRPα) on macrophages, thereby inhibiting phagocytosis [139]. As a result, RBC-NPs demonstrate prolonged circulation times of up to 72 h, substantially surpassing traditional PEGylated nanoparticles. This extended circulation facilitates enhanced tumor accumulation via the enhanced permeability and retention (EPR) effect, improving targeted delivery efficiency [133].

Despite their advantages in tumor targeting, RBC-NPs exhibit limited ability to traverse the BBB [134,140]. To overcome this limitation, Gu et al. [134] engineered RBC-NPs by incorporating the TGN peptide, a BBB-penetrating ligand. This modification increased BBB penetration efficiency to 29.64%, compared to <5% for unmodified RBC-NPs. In an Alzheimer’s disease (AD) model, this system effectively suppressed β-amyloid (Aβ) deposition and attenuated neuroinflammation, demonstrating its potential for central nervous system therapy [134]. While cell membrane coating confers exceptional biocompatibility and immune evasion, challenges remain, including large-scale production, batch-to-batch consistency, and precise functionalization. Future research may explore membranes derived from diverse cell types and integrate specific targeting ligands to further enhance therapeutic precision and versatility [141].

#### 3.4.3. Innovative Applications of Pathogen-Engineered Delivery Systems

Pathogen-inspired delivery systems exploit intrinsic microbial invasion mechanisms, such as immune evasion, cell-specific recognition, and penetration of biological barriers, to achieve enhanced targeting efficiency and effective delivery of macromolecular therapeutics [39]. These features are particularly advantageous for transporting drugs across otherwise impermeable barriers, including the BBB and dense tumor stroma. For example, Bracha et al. engineered *Toxoplasma gondii* to deliver methyl-CpG-binding protein 2 (MeCP2) to the central nervous system, achieving >80% brain distribution in their experimental model, a substantial improvement over the <5% coverage obtained with conventional nanoparticles [135]. Other pathogen-based strategies are under investigation: *Listeria monocytogenes*’s cell-to-cell dissemination has been leveraged for cancer immunotherapy, while adeno-associated virus (AAV) tropism can be tailored to facilitate tissue-specific delivery.

Despite these promising capabilities, clinical translation of pathogen-engineered and other biomimetic systems remains challenging. Major barriers include manufacturing complexity and high production costs, exemplified by the stringent control required for modifications such as chiral functionalization of virus-like nanoparticles, where batch variability may compromise delivery efficiency [14]. Additionally, limited long-term toxicity data and potential immunogenicity present significant concerns for pathogen-derived vectors [135]. Addressing these challenges will be essential for clinical adoption. Interdisciplinary strategies that integrate synthetic biology, materials science, and artificial intelligence offer promising avenues to develop next-generation pathogen-inspired platforms with enhanced safety, reproducibility, and therapeutic efficacy.

### 3.5. Comparative Synthesis of Nanocarrier Design Principles

Across the diverse material classes discussed—lipid, polymeric, inorganic, and biologically derived systems—several overarching design principles dictate therapeutic outcomes. First, a delicate balance of particle size (~50–150 nm) and surface chemistry (neutral to mildly negative charge) is universally required to optimize the EPR effect while evading the mononuclear phagocyte system (MPS). Second, material selection inherently determines targeting capabilities: lipid and polymeric systems excel in biocompatibility and modular ligand conjugation, whereas inorganic carriers uniquely respond to external physical stimuli (e.g., magnetic fields, light). Biologically derived systems provide unmatched immune evasion but face the steepest translational barriers regarding scalability. Ultimately, the rational design of nano-DDS demands matching the specific physicochemical attributes of the nanocarrier to the unique biological barriers of the target pathology.

## 4. Biomedical Applications and Translational Considerations

The therapeutic performance of nano-DDS is ultimately determined by disease-specific biological barriers and clinical requirements. In this section, we primarily focus our discussion on oncology and inflammatory/autoimmune diseases. These conditions present highly complex, dynamic microenvironments—characterized by hypoxia, specific enzymatic overexpression, and dense extracellular matrices—that serve as the most rigorous and extensively researched testing grounds for the targeted and bioresponsive platforms evaluated in this review.

### 4.1. Tumor Treatment

The rapid convergence of nanotechnology and biomedicine has propelled significant advances in nano-delivery systems for cancer therapy [142]. Owing to their unique physicochemical characteristics, these platforms enable efficient drug encapsulation, targeted delivery, and controlled release, addressing limitations associated with conventional therapeutics, such as low bioavailability, nonspecific distribution, and multidrug resistance [68,143]. Multistage delivery systems have emerged as a particularly promising approach. Yuan et al. developed an enzyme-responsive multistage platform consisting of doxorubicin-loaded mesoporous silica nanoparticles encapsulated within liposomes [68]. This system leveraged tumor microenvironmental cues for site-specific drug release, achieving precise treatment of primary colorectal tumors and metastatic lymph nodes. Notably, it increased the 90-day survival rate to 80% in aggressive models, demonstrating the potential of integrating microenvironment-triggered release with hierarchical targeting for metastatic cancers [68].

Targeting the tumor microenvironment represents another critical strategy. Shao et al. highlighted microenvironment-responsive nano-delivery systems capable of overcoming multidrug resistance by exploiting tumor-specific conditions [143]. Enzyme- or pH-responsive carriers, for instance, enable controlled drug release at tumor sites, enhancing therapeutic efficacy while minimizing systemic toxicity [143,144]. Nanotechnology has also advanced cancer immunotherapy. Linderman et al. demonstrated that tumor-responsive nanomaterials could mitigate systemic toxicity and the immunosuppressive tumor microenvironment by enabling precise delivery of immunotherapeutic agents [145]. These platforms exploit dynamic tumor features such as hypoxia and elevated ATP to design responsive delivery systems that potentiate immune checkpoint inhibitors and cytokines, facilitating localized activation of antitumor immunity while minimizing off-target effects. Pulmonary diseases are increasingly targeted by nanomedicine. George et al. reviewed nanoparticle- and nanorobot-based pulmonary delivery strategies, emphasizing their ability to achieve pathogen-targeted therapy with reduced systemic side effects relative to conventional approaches [146].

Erythrocyte-derived delivery systems are emerging as versatile vectors. Della Pelle et al. engineered red blood cell membrane vesicles loaded with siRNA, demonstrating prolonged circulation, passive tumor targeting, and efficient gene silencing, highlighting their potential as biocompatible carriers for gene therapy [147]. Lymph node-targeted strategies have also shown promise. Zhang et al. utilized honeysuckle polysaccharide-loaded exosomes derived from bone marrow mesenchymal stem cells to promote dendritic cell activation and co-stimulation, enhancing lymph node targeting and antitumor immunity [148]. Similarly, tetrahedral DNA-modified extracellular vesicles (EVs) engineered by Wang et al. exhibited enhanced biostability and drug delivery efficiency, achieving notable therapeutic outcomes in oral squamous cell carcinoma models [149].

Despite these advances, key challenges remain, including optimization of targeting efficiency, reduction in immunogenicity, and facilitation of clinical translation. Ongoing technological innovation and mechanistic studies are expected to further improve the efficacy and safety of nano-delivery systems, ultimately enhancing outcomes in cancer therapy.

### 4.2. Inflammation and Autoimmune Diseases

The rapid evolution of nanotechnology has facilitated the development of nano-delivery systems with significant potential for targeted therapy of inflammatory and autoimmune disorders [150,151]. These platforms leverage precise control over drug release, enhanced targeting capability, and responsiveness to pathological microenvironments, collectively achieving high therapeutic efficacy while minimizing systemic toxicity.

In inflammatory arthritis, nanotherapeutic strategies focus on both microenvironment-responsive drug release and modulation of intercellular interactions. Hyaluronic acid-based nanohydrogels, for example, mitigate rheumatoid arthritis-associated bone erosion and inflammation through sustained release of celastrol, suppressing macrophage M1 polarization and inhibiting synovial fibroblast proliferation [151]. MMP-2-responsive micelles enable macrophage-targeted delivery of methotrexate and potentiate quercetin’s anti-inflammatory effects via enzymatic activation, significantly alleviating experimental arthritis symptoms [152]. These studies highlight the capacity of nanocarriers to orchestrate multi-target interventions in complex inflammatory microenvironments.

For inflammatory bowel disease (IBD), redox-responsive nanosystems have emerged as promising therapeutic platforms. Exploiting the ROS-overexpressing microenvironment characteristic of IBD lesions, ROS-scavenging nanoparticles reduce oxidative stress through radical elimination, whereas ROS-triggered carriers achieve site-specific drug release [153]. Such dual-functional designs concurrently suppress inflammatory cascades and minimize systemic exposure, offering innovative strategies for precise IBD management.

Plant-derived nanomedicines further expand the landscape of immunomodulatory interventions. Natural anti-inflammatory and antioxidant phytocompounds encapsulated in nanocarriers exhibit improved stability, targeted delivery, and therapeutic efficacy. For instance, quercetin-loaded micelles enhance macrophage targeting and significantly improve outcomes in rheumatoid arthritis models [152]. Beyond arthritis, plant-based nanoparticles demonstrate potential for modulating chronic inflammation and autoimmune responses, with promising applications in acute inflammatory conditions such as COVID-19 [83]. Despite these advances, clinical translation remains challenging. Critical issues include comprehensive biocompatibility evaluation, scalable manufacturing, stability optimization, and long-term toxicity assessment [83]. Future research will require interdisciplinary integration to refine nanocarrier design, ensuring both efficacy and safety for clinical applications in inflammatory and autoimmune diseases.

In comparing oncology and inflammatory disease models, distinct structure-function-barrier relationships emerge. Tumors typically require nanocarriers that can withstand high interstitial fluid pressure and respond to local hypoxia or acidic pH for penetration. Conversely, therapies for inflammatory diseases (e.g., IBD, arthritis) increasingly rely on redox-responsive or macrophage-targeted mechanisms to scavenge ROS and reverse immune cell polarization. Across both fields, a prominent translational challenge is the paradigm shift from overly complex, multi-stimuli responsive designs toward streamlined, easily scalable formulations that retain high biological specificity without compromising GMP reproducibility.

### 4.3. Translational Hurdles and Clinical Trial Landscape

Despite promising preclinical data, the transition of targeted nano-DDS from the bench to the bedside is severely constrained by translational hurdles. One primary challenge is large-scale, reproducible manufacturing. Complex systems, such as ligand-modified or biomimetic cell-membrane-coated nanoparticles, often suffer from significant batch-to-batch variability, making it difficult to meet the stringent Good Manufacturing Practice (GMP) standards required for clinical trials. Furthermore, comprehensive long-term safety profiles remain a critical bottleneck. Regulatory agencies mandate rigorous toxicological evaluations, yet there is a lack of standardized protocols for assessing the chronic accumulation and immunogenicity of novel materials, such as inorganic alloys and pathogen-derived vectors. Currently, most nano-DDS in clinical trials rely on simpler, robust architectures (e.g., standard PEGylated liposomes), highlighting an urgent need to bridge the gap between academic complexity and industrial scalability.

## 5. Challenges, Opportunities, and Future Perspectives

### 5.1. Overcoming Complex Biological Interfaces

While active and bioresponsive targeting strategies have shown immense potential, navigating the complex human biological interface remains a formidable challenge. The formation of the protein corona in vivo frequently masks targeting ligands, drastically reducing active recognition efficacy. Future research must prioritize the development of dynamic, zwitterionic, or self-shedding stealth coatings that can protect ligands during systemic circulation and expose them exclusively within the target microenvironment. Additionally, combining matrix-modulating agents (e.g., specific enzymes to degrade tumor stroma) with nanocarriers will be essential to overcome physical barriers like elevated interstitial fluid pressure and dense extracellular matrices.

### 5.2. Scalability, Reproducibility, and Regulatory Frameworks

The clinical translation of multifunctional nano-DDS is currently bottlenecked by manufacturing complexities. Transitioning from traditional batch synthesis to continuous microfluidic manufacturing offers a highly promising avenue to ensure tight control over nanoparticle size, drug loading, and batch-to-batch consistency. Concurrently, there is an urgent need for updated regulatory frameworks. Regulatory bodies must establish standardized, universally accepted assays for the quality control of Non-Biological Complex Drugs (NBCDs), specifically tailoring guidelines for the immunogenicity and long-term degradation kinetics of advanced hybrid materials.

### 5.3. Next-Generation AI-Driven Molecular Design

Artificial intelligence (AI) and machine learning (ML) are poised to revolutionize nanomedicine. Future opportunities lie in utilizing predictive algorithms to rationally design lipid and polymer structures, simulating their behavior and pharmacokinetic profiles before in vitro synthesis. However, current AI-driven approaches face notable limitations. These include a heavy reliance on high-quality, standardized in vivo datasets—which remain highly fragmented—and the inherent “black box” nature of complex predictive models, which can complicate regulatory assessment and clinical interpretation. Despite these challenges, by integrating computational materials science with patient-specific genomic and proteomic data, the field can shift from “one-size-fits-all” nano-DDS to truly personalized, intelligent nanotherapeutics capable of executing precise spatiotemporal interventions.

## 6. Conclusions

Targeted nano-drug delivery systems (nano-DDS) represent a transformative paradigm in precision medicine, effectively bridging the critical gap between materials engineering and dynamic biomolecular interactions at the bio-nano interface. By systematically orchestrating passive, active, physical, and bioresponsive targeting strategies, these platforms overcome the intrinsic limitations of conventional pharmacotherapy, offering enhanced bioavailability, spatiotemporal control over drug release, and minimized systemic toxicity. As detailed in this review, the rational design of lipid-based, polymeric, inorganic, and biologically derived nanocarriers has demonstrated significant therapeutic potential, particularly in navigating the complex and dynamic microenvironments of oncology and inflammatory diseases.

However, the transition of these advanced nanotherapeutics from the bench to clinical application remains constrained by significant translational hurdles. Navigating complex biological interfaces—such as overcoming the in vivo protein corona that frequently masks targeting ligands and penetrating dense extracellular matrices—continues to challenge active delivery efficacy. Furthermore, industrial scalability is heavily bottlenecked by manufacturing complexities, batch-to-batch variability, and the critical need for comprehensive long-term safety and toxicological profiles.

Addressing these bottlenecks requires a highly interdisciplinary approach. The integration of continuous microfluidic manufacturing will be crucial for ensuring scalable and reproducible production, while the establishment of updated, standardized regulatory frameworks is imperative for the rigorous clinical evaluation of complex hybrid materials. Ultimately, the advent of artificial intelligence (AI) and machine learning (ML) promises to revolutionize the nanomedicine landscape. By accurately predicting receptor-ligand docking interfaces and simulating pharmacokinetic profiles prior to synthesis, the field is poised to shift from broad-spectrum formulations to highly personalized, intelligent nanotherapeutics tailored to individual patient pathology.

## Figures and Tables

**Table 1:** Rational design and performance of lipid-based nanocarrier systems.

Nanoparticle Type	Targeting Strategy	Biological Model	Key Outcomes	Ref.
PEGylated Liposomes	Passive (EPR) and Enzyme-responsive (MMP-2)	Colorectal cancer primary tumors and lymph nodes (in vivo)	Increased 90-day survival rate from 45% to 80%; improved lymphatic drainage.	[45,68,69,83]
Peptide-modified PEGylated Liposomes	Active (TREM2-targeting peptide)	U87MG Glioma (in vivo orthotopic mouse model)	6.8-fold higher BBB penetration; extended median survival from 28 to 49 days.	[69,70,71,72,73,74,75,76,77]
Solid lipid nanoparticles (SLNs)	Active (Mannose-binding protein-1 receptor)	Toxoplasma gondii infection (in vitro)	82.3% intracellular delivery efficiency; systemic toxicity minimized (HEK293 survival >85%). Understanding of in vivo degradation–pharmacodynamics relationships	[76,79,80,81,82,84,85,86,87,88]
Lipid Prodrug Liposomes	Enzyme-responsive and Immunomodulatory	Colorectal cancer (in vivo)	60% reduction in tumor volume; reprogrammed neutrophils to enhance T-cell infiltration.	[76,78]

**Table 4:** Representative biomimetic nano-delivery paradigms enabling precision drug delivery.

Biomimetic Design Paradigm	Representative System and Components	Key Functional Advantages	Major Limitations and Translational Challenges	Ref.
Bioinspired surface morphology and functional interfaces	Virus-like PLGA nanoparticles with spike structures; intestinal-specific adhesive proteins; pH-responsive shells; insulin	Enhanced intestinal adhesion (8.3-fold vs. smooth particles); improved oral bioavailability of insulin (from 5.2% to 15.7%); prolonged glycemic control (up to 12 h)	Batch-to-batch variability in surface modifications (e.g., chiral functionalization); manufacturing complexity affecting reproducibility	[14]
Biomimetic motility and chemokine-guided targeting	Magnetic nanorobots incorporating CXCR4 chemokine receptors; doxorubicin	Directional migration along SDF-1α gradients; enhanced tumor penetration; ~50% increase in intratumoral drug enrichment with reduced cardiotoxicity	System complexity; challenges in large-scale fabrication and precise external control	[132]
Cell membrane camouflage for immune evasion	Red blood cell (RBC) membrane–coated nanoparticles expressing CD47	Effective immune evasion via “self” signaling; prolonged circulation half-life (up to 72 h); enhanced tumor accumulation through EPR effect	Limited intrinsic ability to cross restrictive biological barriers such as the blood–brain barrier	[133]
Membrane engineering for barrier penetration	TGN peptide–modified RBC membrane–coated nanoparticles	Improved BBB penetration (29.64% efficiency); reduced β-amyloid deposition and neuroinflammation in Alzheimer’s disease models	Added formulation complexity; long-term safety and stability yet to be fully evaluated	[134]
Pathogen invasion–inspired trans-barrier delivery	Engineered *Toxoplasma gondii* (MeCP2 delivery); *Listeria monocytogenes* (cell-to-cell spread); adeno-associated virus (AAV) vectors	Exceptional tissue penetration; >80% brain distribution coverage; efficient macromolecular delivery via natural invasion pathways	High manufacturing cost; biosafety and immunogenicity concerns; limited long-term toxicity data	[135]

## Data Availability

Data sharing is not applicable to this article as no data were created or analysed in this research.
